# The open access and dissemination of predatory journals

**DOI:** 10.5935/0004-2749.2024-1009

**Published:** 2024-05-09

**Authors:** Newton Kara-Junior

**Affiliations:** 1 Ophthalmology Department, Universidade de São Paulo, São Paulo, SP, Brazil

Financial companies profit by charging for market information provided to other corporations, thus benefiting the entire commercial chain. However, when publishers charge to make the knowledge produced by researchers available, access to research is restricted and human development is hindered. Moreover, scientific reviewers, who typically work without pay for the benefit of the public, are also subjected to abuse. Open access refers to free access to published scientific research. It operates under the assumption that scientific knowledge is public and, as a result, should be accessible to all individuals^(1)^.

For this reason, the scientific community vigorously supports open access, which is becoming more prevalent. However, the popularity of open access has led to the emergence of new challenges. Many scientific journals have started charging researchers to publish studies to maintain open access and cover professional and expensive editorial process costs. This practice is difficult to regulate, and opportunistic groups have realized a financial opportunity that has given rise to predatory journals.

Predatory journals prioritize profit over the dissemination of scientific knowledge. In general, these journals publish papers without subjecting them to peer review. The authors just have to pay for the article to be published. These journals typically promote inaccurate details regarding their indexing, impact factor, and editorial procedures while using names that resemble reputable journals to confuse and attract authors. Charging fees for publishing articles has led to the proliferation of predatory journals.

Journals, regardless of being predatory or not, serve the practical purpose of disseminating knowledge, making them valuable for researchers aiming to share their studies. Predatory journals disseminate research without adequate peer review, inundating the scientific community with unreliable information. Today, readers face the challenge of discerning reliable information from the vast amount available, as there is a presence of misinformation or fake news in both scientific and everyday contexts.

After the consolidation of open access, the international scientific community may focus on eliminating publication fees. Public funding agencies primarily cover most publication fees from research grants for researchers and their projects. Funding agencies can sponsor high-quality scientific journals directly as a solution. This situation would decrease reliance on intermediaries in the scientific community and eliminate financial support for predatory journals.
